# Evaluation of a new portable 1-lead digital cardiac monitor (eKuore) compared with standard base-apex electrocardiography in healthy horses

**DOI:** 10.1371/journal.pone.0255247

**Published:** 2021-08-03

**Authors:** Valentina Vitale, Tommaso Vezzosi, Rosalba Tognetti, Carlotta Fraschetti, Micaela Sgorbini

**Affiliations:** Department of Veterinary Sciences, University of Pisa, San Piero a Grado, Pisa, Italy; University of Perugia, ITALY

## Abstract

Recently, the use of smartphone ECG devices has been reported in humans and animals. Nevertheless, as the electrodes of these devices are inseparable, they can create only a precordial reading in veterinary species. Thus, although the smartphone ECG devices are considered valuable as a screening tool for the detection of some common arrhythmias, they are not always a reliable method for the measurement of the duration of the electrical deflections. The objectives of this study were to evaluate the feasibility of a novel smartphone ECG device, to report the readings obtained recorded simultaneously with a reference ECG system, and to compare the heart rate and duration of cardiac deflections obtained with the two methods. A total of 28 healthy mares of different breeds and age were included in this study and ECG recordings were obtained simultaneously with a reference ECG telemetry system with surface electrodes attached to the skin with alligator clips using a standard base-apex system and a smartphone ECG device with electrodes positioned alternatively with a standard and a modified base apex derivation. All the recordings obtained were considered acceptable for interpretation. An excellent agreement was found between the two methods for the evaluation of heart rate and polarity of cardiac deflections. No differences regarding number, duration and percentage of artifacts were found. This technology could become a valid diagnostic tool in the cardiological assessment of horses, in particular on the field.

## 1. Introduction

In horses, surface ECG is considered the gold standard for the diagnosis of arrhythmias [1]. The procedure requires an ECG machine, cables and electrodes, thus is not always practical to use in the field [2]. Recently, the use of smartphone ECG (spECG) devices has been reported in humans and animals and good accuracy for evaluation of heart rate and rhythm has been described [3–11]. For the acquisition of SpECG readings in humans, the patient usually touches the electrodes of the device with the right and left hands, creating a recording similar to lead I in a 12-lead ECG [7]. In veterinary species, as the electrodes are inseparable with the devices currently used, they need to be positioned both on the thorax creating only a precordial reading. [3] Therefore, although the spECG devices are considered valuable as a screening tool for the detection of some common arrhythmias in horses, they are not always a reliable method for the measurement of the duration of the deflection on the electrical baseline [3,12]. Furthermore, the interpretability of the electrical waves was slightly different when the device was placed on the left or right side of the thorax [3].

The objectives of this study were to (a) evaluate the feasibility of a novel smartphone-based ECG device, (b) report the readings obtained with a standard and modified base-apex placement of the device’s electrodes recorded simultaneously with a reference ECG system (rECG), and (c) compare the heart rate and duration of cardiac deflections obtained with the spECG with the rECG. The hypothesis is that good agreement would be found for both the HR and the duration of deflections recorded with rECG and spECG with both standard and modified base-apex placement.

## 2. Materials and methods

### 2.1. Animals

A total of 28 healthy mares owned by–masked for review–were included in the study that took place between January and June 2020. The mares were considered healthy based on history, physical examination, ECG and echocardiography. The age ranged between 4 and 20 years old (median 8.5 years), the BW between 430 and 630 kg (median 522 kg) and the BCS score between 4 and 5/9 (median 4.5). They were 21/28 Standardbred (75%), 6/28 Thoroughbred (21%), and one Anglo-Arab (4%). The research protocol was approved by the Institutional animal care and use committee of the University of Pisa (45965/2016).

### 2.2. ECG acquisition

For the study protocol each mare was restrained in a stock and, after 5 minutes of acclimatization period, two recordings of 30 seconds were realized simultaneously with the rECG and the spECG.

The rECG (Televet 100, Engel Engineering Service GmbH, Germany) was acquired using surface electrodes attached to the skin with alligator clips using a standard base-apex system [13]. The yellow positive electrode (left arm) was positioned on the left precordial area, the red negative electrode (right arm) was positioned on the right jugular groove in the middle third of the neck, and the neutral and ground electrodes (black and green) was placed at the tip of the left shoulder and at the level of the sternum, respectively. Alcohol was applied on the clips to ensure electrical contact. The ECG tracings were printed with a paper speed of 25 mm/s with an amplitude of 10 mm/mV. A sampling rate of 500 Hz was used for standard ECG acquisition, with a 100 Hz low-pass filter and a 0.05 Hz high-pass filter.

**Fig 1:**
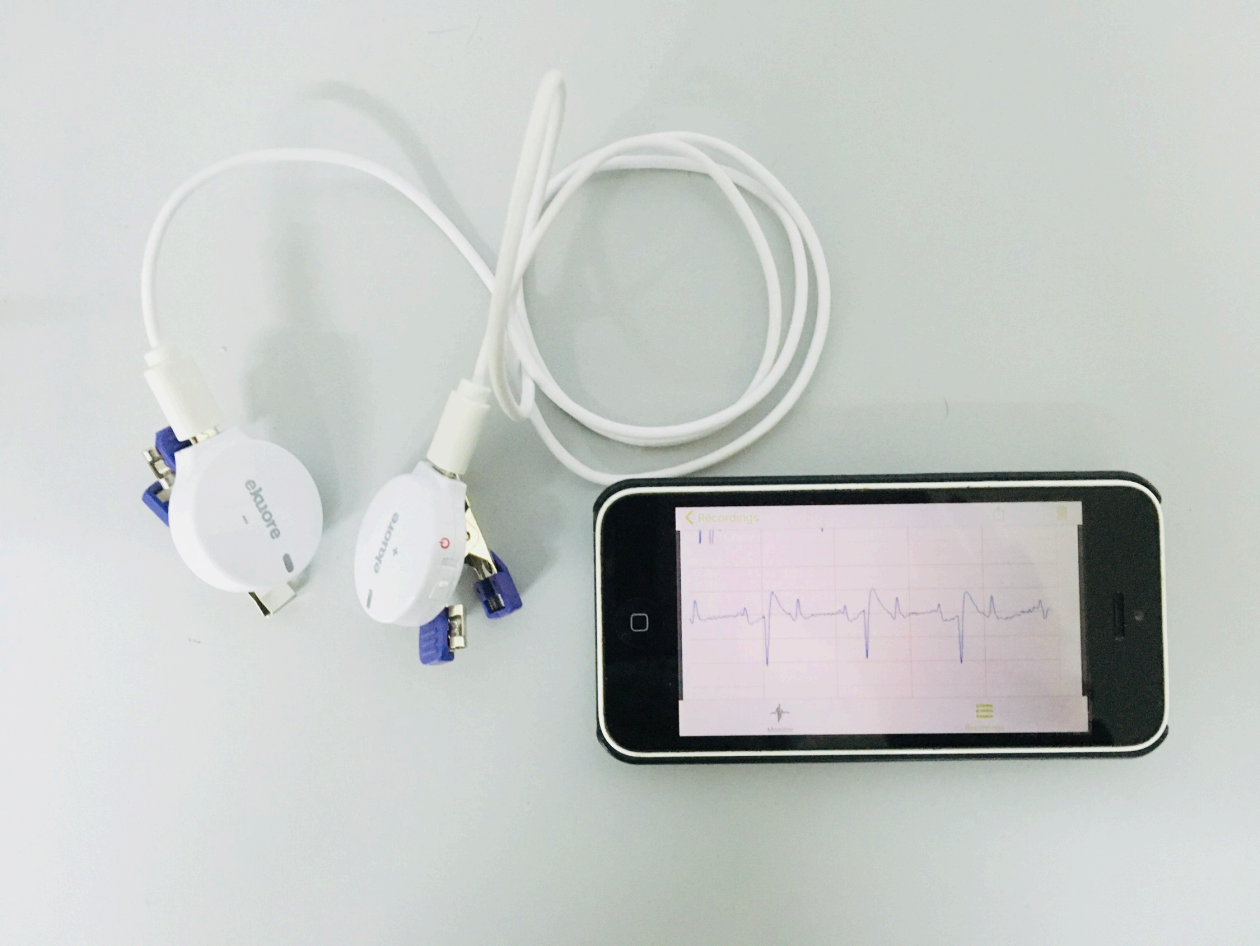
The device consists in two electrodes with clips connected by a micro-USB cable and a smartphone with the app eKuore ECG (Chip Ideas Electronics SL, Spain).

The spECG device was applied using a single-lead bipolar ECG (1-lead ECG eKuore, Chip Ideas Electronics SL, Spain) and its smartphone application (eKuore ECG, Chip Ideas Electronics SL, Spain) (Fig 1).

**Fig 2:**
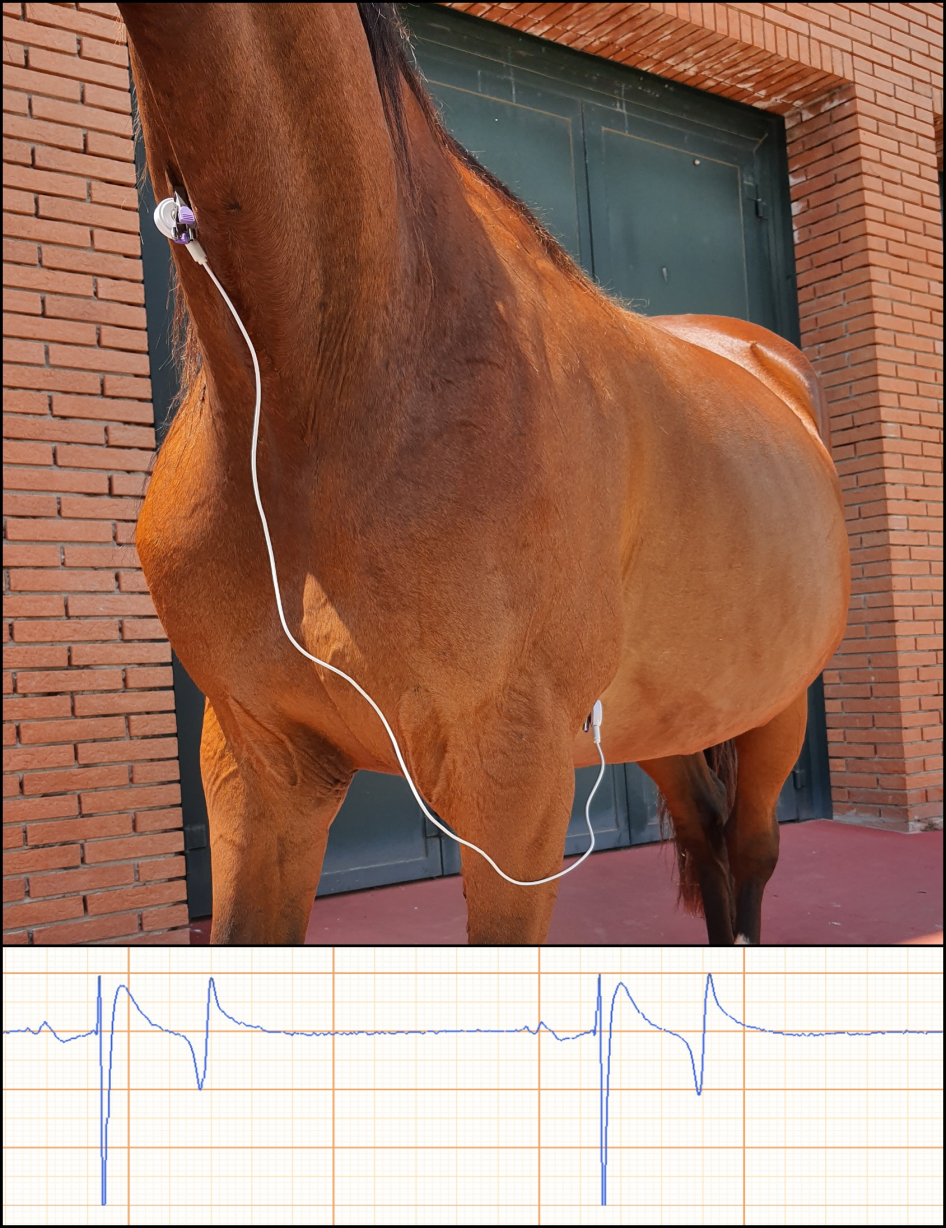
Standard base-apex (SBA) placement of the smartphone ECG (spECG) electrodes.

The tracings were recorded with an iPhone 5S (Apple, USA), automatically digitalized by the device, archived as PDF and printed with a paper speed of 25 mm/s and an amplitude of 10 mm/mV. The two electrodes were applied with two different methods previously described: a standard (SBA) and a modified (MBA) base-apex derivation [12,14]. A first 30 seconds-recording was obtained with the rECG and the SBA configuration and, immediately after repositioning the electrodes of the spECG, another 30 seconds-recording was obtained again with both rECG and the MBA configuration of the spECG. For the SBA position the positive electrode was placed on the left precordial area, and the negative electrode was placed on the right jugular groove in the middle third of the neck (Fig 2).

**Fig 3:**
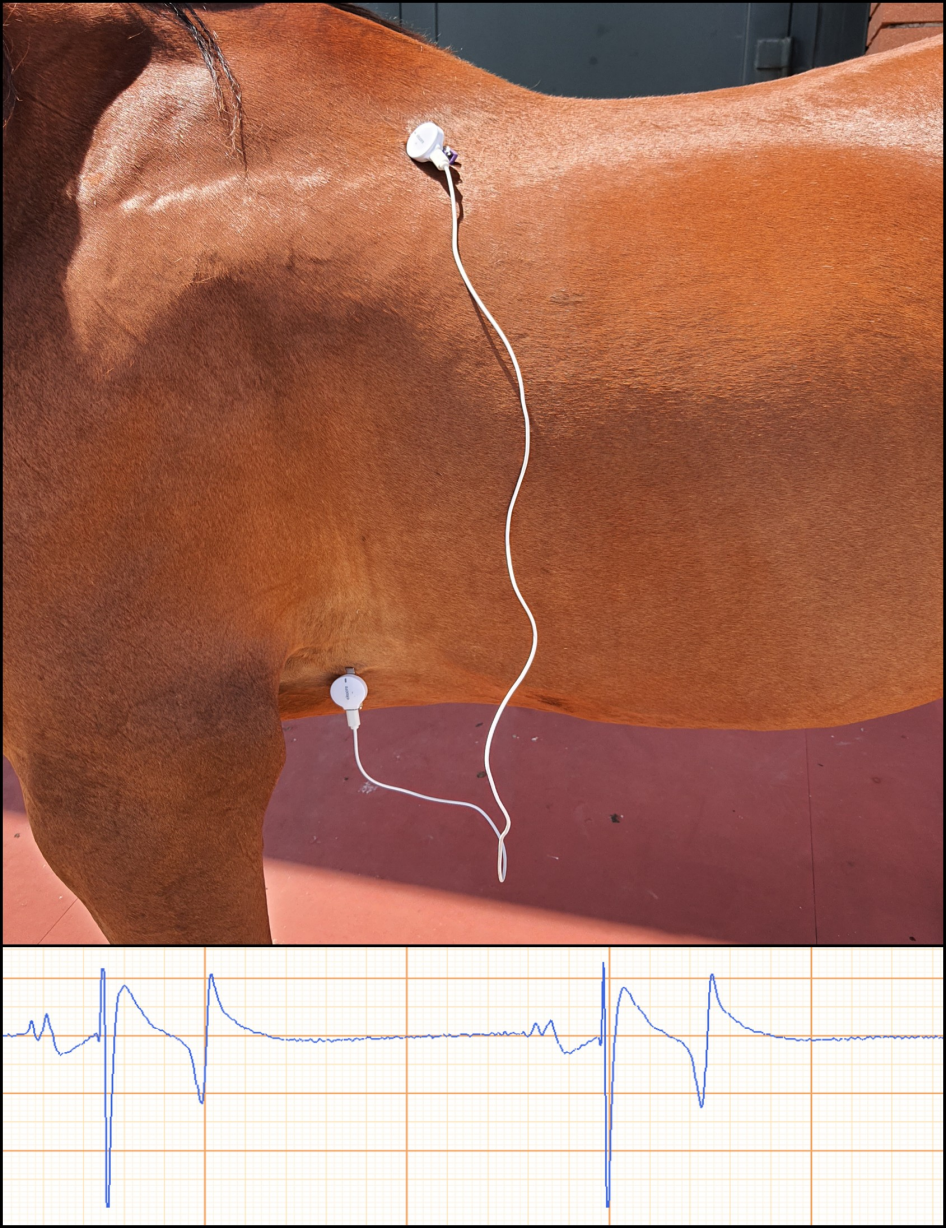
Modified base-apex (MBA) placement of the smartphone ECG (spECG) electrodes.

For the MBA position the positive electrode was maintained in the same position, while the negative electrode was applied on the left side of the withers (Fig 3).

Alcohol was applied on the skin before the placement of the electrodes to ensure electrical contact. The same operator (CF) was responsible of the placement and recordings of both the rECG and spECG in all horses.

### 2.3. ECG evaluation and measurements

All smartphone ECG tracings were evaluated by an experienced operator (TV) blinded to the study to assess whether the tracings were acceptable or not for interpretations. Tracings were considered acceptable for interpretation if baseline artifacts were absent for at least the 80% of the recording. Baseline artifacts were defined as ECG segments in which P-waves and/or QRS complexes could not be identified. The same operator performed also the ECG measurements on all tracings, using lead I of the rECG and the only available lead of the spECG. The following variables were measured for both rECG and spECG in each recording session: mean heart rate (HR; bpm), P-wave duration (milliseconds [ms]), PQ interval (ms), QRS complex duration (ms), and QT interval (ms). Heart rate (HR) was calculated counting the number of QRS complexes on 15 cm and multiplying the result by 10 for the spECG (paper speed of 25 mm/s). Mean HR was calculated as the mean value of three independent HR calculations from three different areas on the ECG tracings. The duration of each wave or interval was calculated as the average of the measurements of three randomly selected heartbeats.

The polarity of P-waves and QRS complexes, as well as the presence/absence and duration (ms) of artifacts were also evaluated.

### 2.4. Statistical analysis

Analysis was performed only with paired ECG tracings that were considered acceptable for interpretation and the rECG was considered the reference method. Cohen’s k test was used to calculate the agreement between the spECG and the rECG for the classification of the polarity of P-waves (positive, negative or biphasic) and QRS complexes (positive or negative). The k coefficient was interpreted as follows: k < 0.01: null agreement; 0.01< k < 0.20: poor agreement; 0.21 < k < 0.40: modest agreement; 0.41 < k < 0.60: moderate agreement; 0.61 < k < 0.80: good agreement; 0.81 < k < 1.00: excellent agreement. If the contingency table reported one or more values equal to zero, Cohen’s k could not be calculated and therefore in these cases the percentage of agreement was used instead.

Differences in the prevalence of baseline artifacts on spECG and rECG were evaluated using McNemar test. This test compares the observed frequencies (presence/absence of artifacts on spECG vs rECG) with those expected.

Wilcoxon matched-pairs signed ranks test for non-paired data was used to verify the differences in number, duration and prevalence of artifacts. The Bland Altman test was used to evaluate the differences in duration of the electrical defections with spECG and rECG and 95% bias and limits of agreement were calculated for HR, P-wave duration, PR interval, QRS complex duration and QT interval.

Statistical analysis was performed with a commercial software (Microsoft Excel, 2011; GraphPad Prism 6, USA) and a P value < .05 was set as significant.

## 3. Results

### 3.1. Animals and feasibility

Within the subjects included in the study, all 28 mares showed a normal sinus rhythm, one of them (3.6%) presented a single episode of second-degree atrioventricular block according to rECG.

Among the smartphone ECG tracings, all the 56 recordings obtained from the 28 mares with the two methods of electrodes placement were considered acceptable for interpretation, thus no one was excluded from the statistical analysis.

### 3.2. Heart rate

With the first 30 seconds of recording the median HR was 60 bpm with both the rECG and the spECG with the SBA configuration. The bias between the HR measured manually on rECG and SBA configuration of the spECG was 4 bpm (95% CI: -5 to 12 bpm). The median HR obtained with the successive 30 seconds of recording was 50 bpm with both the rECG and the MBA configuration of the spECG. The bias between the HR measured manually on rECG and spECG with MBA configuration was 1 bpm (95% CI: -5 to 3 bpm).

### 3.3. Cardiac electrical deflections

The median duration of the electrical deflections measured on the tracings from the rECG and the two electrodes configurations of the spECG are reported in Table 1, along with the bias calculated from their comparison.

**Table 1:** Duration of cardiac deflections.

Deflections	rECG	spECG with SBA configuration	Bias (95% CI)	rECG	spECG with MBA configuration	Bias (95% CI)
P-wave (ms)	140	140	0 (-22 to 22)	135	120	1 (-30 to 32)
PQ interval (ms)	320	340	-1 (-46 to 16)	320	320	1 (-40 to 41)
QRS complex (ms)	120	120	-4 (-27 to 19)	120	120	1 (-25 to 27)
QT interval (ms)	400	420	-11 (-45 to 23)	420	440	-13 (-43 to 16)

Median duration of each cardiac electrical deflection measured on the tracings from the reference electrocardiography (rECG) and the smartphone electrocardiography (spECG) with both the standard base-apex (SBA) and the modified base-apex (MBA) configurations. Bias with 95% of confidence intervals (CI) obtained from the comparison of the rECG and each spECG configuration are also reported.

**Fig 4:**
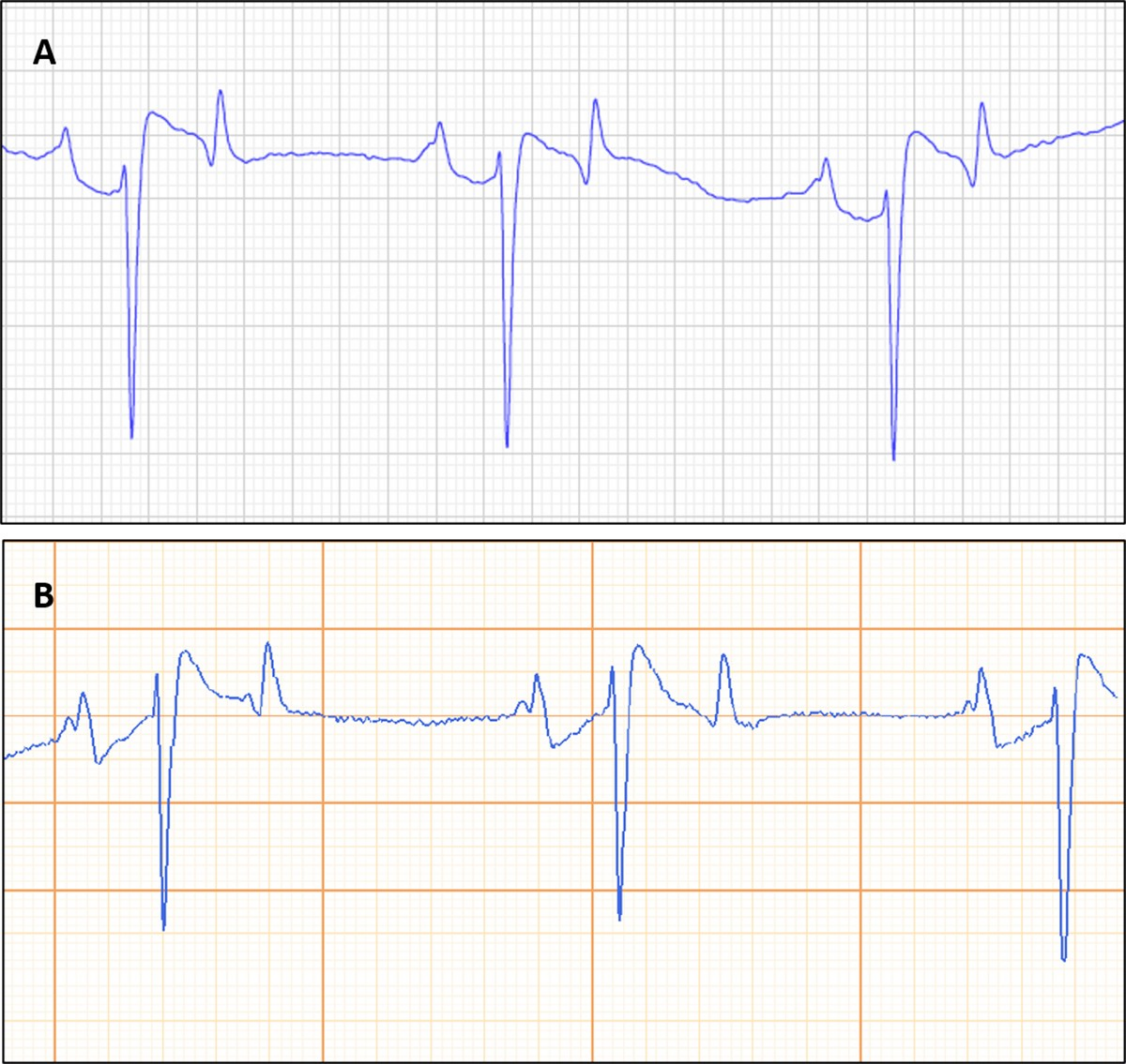
Side-by-side example of a tracing obtained simultaneously with reference ECG (rECG; A) and smartphone ECG (spECG; B) showing the same polarity of the cardiac deflections. Paper speed = 25 m/s; 10 mm/mV.

P-wave polarity was positive in all recordings with both the rECG and spECG, independently from electrode configuration. Therefore, an excellent agreement was found when comparing the rECG and the spECG for the evaluation of the polarity of P-waves. QRS complexes had negative polarity in all recordings with both the rECG and spECG, independently from electrode configuration. Thus, the agreement between the rECG and the spECG for the assessment of the QRS complex polarity was excellent (Fig 4).

### 3.4. Artifacts

**Fig 5:**
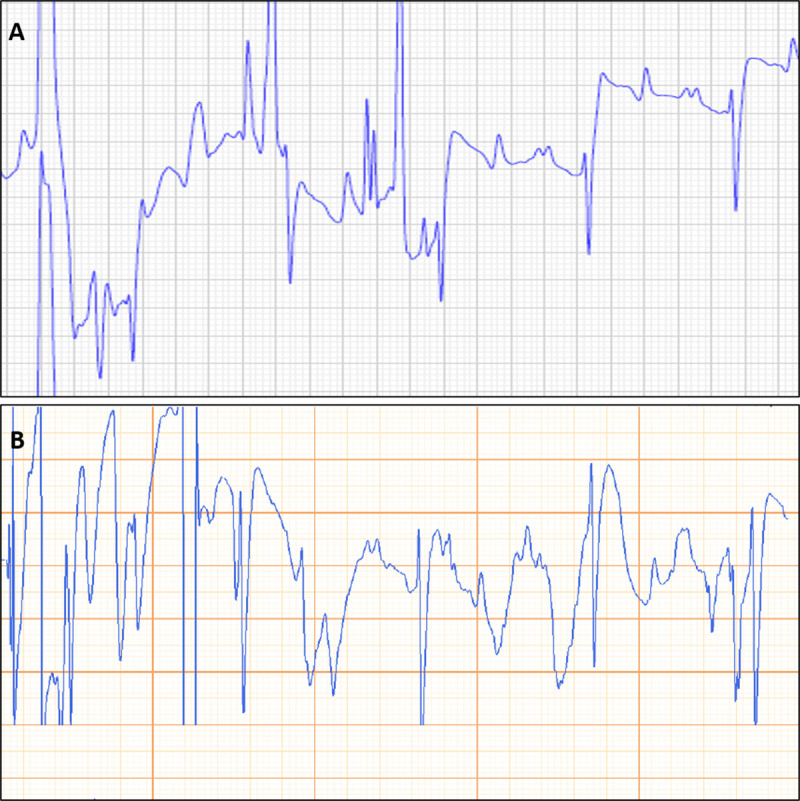
Side-by-side example of a tracing with baseline artifacts obtained simultaneously with reference ECG (rECG; A) and smartphone ECG (spECG; B). Paper speed = 25 m/s; 10 mm/mV.

For the SBA electrodes placement of the spECG, 16 out of 28 tracings (57%) presented baseline artifacts. The median duration of the artifacts was 2500 ms (range: 400–10000 ms), corresponding to a median of 8.3% of the total duration of each recording. On the rECG recorded simultaneously, 21 out of 28 tracings (75%) presented baseline artifacts with a median duration of 2000 ms (range 500–7200 ms), corresponding to a median of 6.6% of the total duration of the recordings (Fig 5).

No differences in the frequency of artifacts between the rECG and the spECG were detected (P = 0.131). No differences between the rECG and the spECG regarding number (P = 0.211), duration (P = 0.121) and percentage (P = 0.272) of artifacts were found.

For the MBA electrodes placement of the spECG 18 out of 28 tracings (64.2%) presented baseline artifacts. The median duration of the artifacts was 7400 ms (range: 800–14000 ms), corresponding to a median of 24.6% of the total duration of each recording. On the rECG recorded simultaneously, 20 out of 28 tracings (71.4%) presented baseline artifacts with a median duration of 4350 ms (range 600–11800 ms), corresponding to a median of 14.5% of the total duration of the recordings. No differences in frequency of artifacts between the rECG and the spECG were detected with the Fisher’s exact test (P = 0.450). No differences were found between the rECG and the spECG regarding number (P > 0.999), duration (P = 0.638) and percentage (P = 0.627) of artifacts.

## 4. Discussion

This study describes the use of a novel smartphone-based ECG device in healthy horses, comparing the tracing with a reference ECG system. The used eKuore device is commercialized to monitor cardiac activity in veterinary medicine. It is constituted of two small electrodes that communicates wirelessly to an app available both on iOs or Android. The use of the device was reliable in obtaining ECG recordings in all the horses included in this study. All the tracings obtained were judged interpretable, despite the presence of occasional short-lived artifacts, compatible with motion or muscle tremor artifacts. This is in line with results reported in horses with other smartphone ECG devices [2,3,12,15–17].

In our study the spECG was reliable in manually measuring HR in horses, similarly to what has been previously described by others [2,3,17]. The elevated HR obtained in this study should be attributable to stress or elevated sympathetic tone of the horses included. To reach the stock where the study protocol took place, they were hand-walked for approximatively 200 meters and the 5-minutes period of acclimatization was probably too short to allow the return of the HR to baseline. This finds confirmation by the reduction of the HR from 60 bpm to 50 bpm in the second session of recordings.

The spECG was found to be reliable also in the measurement of the duration of the ECG waves with minimal and not clinically relevant differences when compared with the rECG. Although the duration of QRS complexes and QT segments has no clinical value in horses, this is in line with previous studies in dogs, horses, and cows [2,4,5,8,11,12,15].

Notably, excellent agreement was also found for the analysis of the polarity of P-waves and QRS complexes. In the tracings of all the horses of this study P-waves and QRS complexes showed the same polarity on spECG and rECG. These findings are in line with what has been already reported in dogs [4] but, especially regarding the P-wave polarity, not with what has been observed in cattle, horses and cats [2,3,5,18]. Furthermore, in horses P-waves were found to be more noticeable when the precordial electrodes of the spECG were placed over the right side of the thorax [3], while with the device reported here results of spECG were superimposable to those of the rECG (Fig 4). The greater reliability of the digital cardiac monitor system used in this study, compared with previous studies, could be useful in the diagnosis of supraventricular arrhythmias, in which the morphology and the polarity of P-waves are important [1,19].

The positioning of the electrodes is not strictly defined in horses and can be adapted according to the circumstances [20], however the base-apex lead system is widely accepted [21]. With this study we compared the recordings obtained with the rECG and the spECG with both SBA and MBA positioning and no significative differences were found on all the values analyzed. Especially in the field with only one operator performing the ECG, the MBA positioning can be more practical as the electrodes will remain all on one side of the horse. Furthermore, the SBA system is unsuited for recordings during exercise, since electrodes are more prone to create movement artefacts and fall off. [21,22] Moreover, an additional advantage of this device compared to the previous spECG described in horses is that the precordial leads should have been maintained in place by the operator [2], while with the system described in this study that is not necessary, allowing longer recordings and keeping the operators in a safer position.

One of the main limitations of this study is the small number of horses included and the fact that they were all healthy, thus no arrhythmias could be evaluated. Furthermore, the HR recorded was above the normal range in most of the cases, probably due to the short acclimatization period. Thus, the results obtained may not be valid at lower HR and should be confirmed with further studies. In addition, the ECG tracings were always obtained by a single operator and they were blindly evaluated by another single operator, thus interobserver variability was not assessed.

In conclusion the spECG with both electrodes positioning is a reliable method to record good-quality single-lead ECG tracings in horses. It has been demonstrated reliable in evaluating HR, measuring the duration of cardiac deflections and determining the polarity of P-waves and QRS complexes. This technology could become a valid diagnostic tool in the cardiological assessment of horses, in particular on the field. As the device is user-friendly and easy to apply, it could be interesting to evaluate if the recordings can be performed also by lay people instructed by veterinarians, as it has already been described in dogs [23]. Should the quality of the recordings remains good and the tracings are judged interpretable, that would allow a leap forward for the telehealth in veterinary medicine and prompt diagnosis and treatment, as it already happens in humans [24]. Further studies are needed also to evaluate the diagnostic accuracy of the device in the detection of cardiac arrhythmias in horses.
